# Fabrication of Novel Crosslinking Carboxylic Styrene-Acrylate Latices as Binders for Exterior Flexible Facing Tiles

**DOI:** 10.3390/molecules28176249

**Published:** 2023-08-25

**Authors:** Yue Lu, Jingke Wei, Haojie Jin, Liming Tang

**Affiliations:** Key Laboratory of Advanced Materials of Ministry of Education of China, Department of Chemical Engineering, Tsinghua University, Beijing 100084, China

**Keywords:** styrene-acrylate latex, mechanical performance, binder, exterior flexible facing tile, mineral powder

## Abstract

To overcome the shortcomings of the temperature sensitivity of exterior flexible facing tiles (EFFIs), a series of crosslinking carboxylic styrene-acrylate (SA) latices were prepared via the semicontinuous seed emulsion polymerization of glycidyl methacrylate (GMA), methacrylic acid (MAA), acrylic acid (AA), butyl acrylate (BA), and styrene (St), and were applied as binders to fabricate EFFTs with mineral powder. The obtained latices exhibited Bragg diffraction because of the narrow particle size distribution. Owing to the low dosage of emulsifiers and the crosslinking reaction between the epoxy group and the carboxyl group, the latex films displayed excellent water resistance, with water adsorption as low as 7.1%. The tensile test, differential scanning calorimeter (DSC) test, and dynamic mechanical analysis (DMA) indicated that at a GMA dosage of 4–6% the latex films had high mechanical strengths, which remained relatively stable in the temperature range of 10 to 40 °C. The optimal AA dosage was found in the range of 2 to 3%, at which the wet mixture exhibited good processability, conducive to forming an EFFT with a compact microstructure. Using the optimal SA latex, the obtained EFFT displayed a series of improved performances, including low water absorption, high mechanical strength, and stable self-supporting ability over a wide temperature range, exhibiting the application potential in the decoration and construction industries.

## 1. Introduction

The booming development of the construction industry and the enormous requirements for building packaging materials have had a negative impact on the environment. The overuse of natural resources as raw building materials and the disposal of waste materials via burning or landfilling cause serious environmental damage and pollution [1]. Although ceramic tiles are commonly used for decoration, their high density, poor cracking resistance, huge energy consumption, and heavy environmental burden are not recognized by the public [2,3]. In contrast, as a new type of environmental protection building decoration material, an exterior flexible facing tile (EFFT) has the advantages of being light weight, flexibility, impact resistance, a rich texture for customization, high safety, low environmental impact, and recycling, becoming a potential alternative to a ceramic tile [4]. Typically, an EFFT is made from construction waste (cement, stone powder, etc.) and a polymer binder by creating a wet mixture (slurry), filling the mold, curing the wet mixture, and demolding [5].

Water-based styrene-acrylate (SA) latex is widely used in building coatings [6,7] and adhesives [8,9] due to its good performance, low cost, safety, and environmental friendliness [6,7,8,9,10,11]. As of today, the commercial SA latices directly applied in EFFTs have the problems of hardening in winter and softening in summer because of their non-crosslinked linear polymer chains, seriously impacting service life at low and high temperatures. Crosslinking provides a powerful means of modifying the structures and properties of linear polymers [12]. The drawbacks, such as low water resistance, and the great change in mechanical properties with environmental temperature can also be overcome using crosslinking strategies [13,14] because, at highly elastic states, crosslinked polymers possess mechanical properties insensitive to temperature. Among various crosslinking strategies, a self-crosslinking pathway has been highly desired, which includes a siloxane self-crosslinking pathway [15,16], epoxy self-crosslinking pathway [17,18], and acrylamide derivative (such as N-methylol acrylamide) self-crosslinking pathway [19,20] because of the easy operation and mild reaction condition. For crosslinking polymer binders used in EFFTs, it is also necessary to carefully modulate its glass transition temperature (Tg) to ensure that the Tg is consistent with the service temperature of the EFFT. 

Mixing waste tire rubber materials with cement concrete materials has become an effective method to improve the performance and durability of concrete. However, poor compatibility between organic and inorganic materials induces a significant decrease in the strength and modulus [21,22]. In order to solve this problem, various surface modification strategies of rubber particles have been proposed, including surface treatment with a NaOH solution [23,24], bonding with a silane coupling agent [25,26], and modification with a polymer latex [27,28,29,30]. Surface modification with a polymer latex bearing functional groups, such as carboxylate polystyrene latex [27], polycarboxylate latex [28], polyvinyl alcohol latex [29], and vinyl acetate copolymer latex [30], not only solves the problem of poor compatibility but also forms a continuous film with which to wrap mineral powders [31], effectively increasing the strength of the composite materials [27]. Similarly, in the production of an EFFT, SA latex can intersperse between inorganic mineral powders and act as a binder after forming a film, drastically increasing the strength of an EFFT. For example, Zhang and coauthors described a method to prepare an EFFT using a GMA- and KH 570-modified water-based polymer emulsion as the binder [32]. In their work, an amine curing agent must be added to realize the post-crosslinking of the epoxy groups, complicating the preparation process of an EFFT. It remains a challenge to develop a simple and efficient method to prepare crosslinking SA latex as the binder of an EFFT.

Herein, we undertook a semicontinuous seed emulsion polymerization of glycidyl methacrylate (GMA), methacrylic acid (MAA), acrylic acid (AA) butyl acrylate (BA), and styrene (St) to prepare crosslinking carboxylic SA latices, which acted as binders for forming an EFFT with excellent processability, low water absorption, and, in particular, stable mechanical strength at a wide service temperature. The water resistance, mechanical properties, and Tg of the latex films with different dosages of GMA were investigated and discussed at a molecular level. The effects of the dosage of AA on the processability of a wet mixture formed of latex, mineral powder, and water, as well as the microstructure of an EFFT, were further investigated and discussed. Overall, our investigation showed that the modulation of polymer chain softness and the introduction of the crosslinked structure are effective in generating high-performance latex films suitable for forming a temperature-insensitive EFFT. 

## 2. Results and Discussion

### 2.1. Preparation and Characterization

In order to prepare crosslinking carboxylic SA latices that could be used as binders to fabricate an EFFT with mineral powder, the semicontinuous seed emulsion polymerization of GMA, MAA, AA, BA, and St was conducted at 80 °C using ammonium persulphate (APS) as the initiator, ammonium sulfate allyloxy nonphenoxy poly(ethyleneoxy) (10) ether (DNS-86) and octylphenol polyoxyethylene ether (OP-10) as the emulsifiers, and NaHCO_3_ as the pH regulator. The monomer feeding formula is summarized in Table 1, wherein the dosage of GMA varied from 0 to 8%, the dosage of AA is fixed to 1%, and the dosage of MAA changed from 0 to 3.8% from sample 1 to sample 5 to ensure that the stoichiometric ratio of the epoxy group and the carboxyl group is close to 1:1, except for sample 1 without GMA and MAA. To make sure that the obtained polymers have relatively low Tgs, the dosage of BA is fixed to a high value (65%) for all of the samples. Based on the total weight (100%) of all of the monomers, the dosage of St is calculated, which varied from 34% to 22.2% from sample 1 to sample 5. Unless otherwise indicated, all of the monomer dosages are weight percentages. The weight ratio between all of the monomers and water is 1:1, so that the solid content is 50% for these samples.

Before conducting the emulsion polymerization, the monomer mixture was dropped into the aqueous solution of the emulsifiers under vigorous stirring, forming the pre-emulsion. After initiating the seed emulsion polymerization of 5% of the pre-emulsion at 80 °C using APS, the remaining pre-emulsion was slowly dropped into the reaction flask and the polymerization was continued at 80 °C for a desired period. Afterwards, the polymerization was further conducted at 60 °C in the presence of tert-butyl hydroperoxide (TBHPO) and sodium formaldehyde sulfoxylate (SFS) as the redox initiator to make sure that almost all of the monomers participated in the polymerization. Using this process, the monomer conversion was determined to be in the range of 98% to 99% for all of the samples.

The effects of GMA dosage on the coagulation fraction of the polymerization process, the particle size, and the Zeta potential of the SA latices were investigated. Although the dosage of the emulsifiers is relatively low (1.2%), the coagulation fractions are all quite low (ranged from 0.069% to 0.213%) in these samples, as indicated in Table 2. This is because both a polymerizable emulsifier (DNS-86) and non-ionic emulsifier (OP-10) were used in this process, which separately provided electrostatic repulsion and steric repulsion to stabilize the latex particles. Owing to the low dosage of the emulsifiers, the latex particle sizes are relatively large (at several hundred nanometers), with a low polydispersity index (PDI) (0.100–0.172). In addition, all of the obtained latices have a Zeta potential below −45 mV, indicating their high stability. 

**Figure 1:**
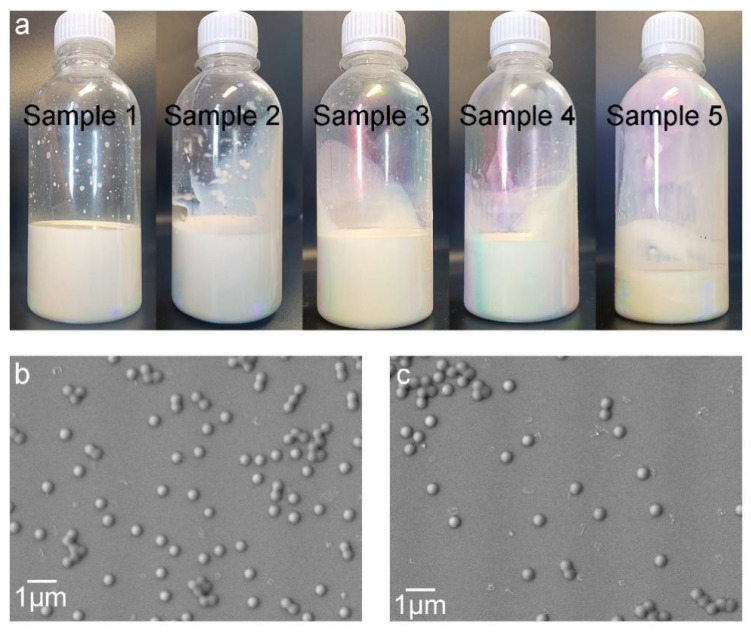
Photographs and SEM images of SA latices. (**a**) Photographs of SA latices (sample 1 to sample 5 from left to right); (**b**) SEM image of SA latex particles with 4% GMA; and (**c**) SEM image of SA latex particles with 6% GMA.

Photographs of the resulting latices are shown in Figure 1a. The appearance of the latices presents a charming color, implying short-range ordered liquid photonic crystals formed in the latices and diffraction appearing based on Bragg’s law of diffraction [33]. When the concentration of latex exceeded the critical volume fraction, good sphericity and uniformity were conducive to the infinite approximation between latex particles to form molecular crystals via self-assembly [34]. Figure 1b,c depict the SEM images of the latex particles with GMA dosages of 4% and 6%, respectively, which present the good sphericity of the particles and the narrow dispersity of the particle size. Using Nano Measure 1.2.0 software, statistic collection based on 126 dispersed latex particles in the SEM image (see Figure S1) of sample 3 gives an average particle size of 0.40 μm, together with a maximum particle size of 0.47 μm and a minimum particle size of 0.35 μm.

**Figure 2:**
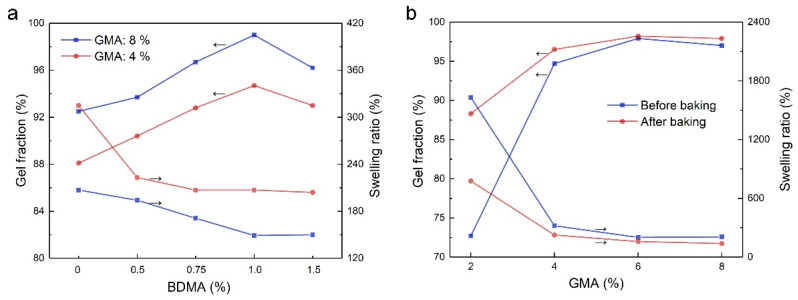
The gel fractions and swelling ratios of SA latex films. (**a**) Influences of BDMA dosage on the gel fraction and swelling ratio of SA latex films; (**b**) influences of GMA dosage on the gel fraction and swelling ratio of SA latex films.

After completing the polymerization process the pH was adjusted to 7–8 with ammonia water, and a suitable amount of *N*,*N*-dimethylbenzylamine (BDMA) was added to the latices, which catalyzes the crosslinking reaction between the epoxy group and the carboxyl group [35] in the baking process. To form SA latex films, the latices were cast on a polytetrafluoroethylene panel and baked at 100 °C for 45 min to complete the crosslinking reaction. The influences of BDMA dosage on the gel fraction and swelling ratio of the films were investigated. From the results in Figure 2a, the gel fraction increases firstly as BDMA dosage until the highest gel fraction is obtained at a BDMA dosage of 1% for the two latex films with GMA dosages of 4% and 8%. Meanwhile, the swelling ratio decreases gradually with an increase in BDMA dosage to reach a stable value at a BDMA dosage of 1%, corresponding to the optimal BDMA dosage. The gel fraction and swelling ratio of the films before and after baking were also determined. From the results in Figure 2b, it is obvious that the gel fraction of the films is already high before baking, especially for those with high GMA dosages. Therefore, the crosslinking reaction between the epoxy group and the carboxyl group had taken place in the polymerization process, and it progressed further in the baking process to improve the gel fraction. The gel fraction increases rapidly with GMA dosage until a stable gel fraction is arrived at with a GMA dosage of 6%, at which point a stable swelling ratio could be obtained. On the contrary, for sample 1 without GMA, the corresponding film dissolved completely in the testing solvent. 

The FT-IR spectra of the SA latex films (sample 4) obtained before and after baking (100 °C) are shown in Figure S2. It is noted that there is no significant difference between these two spectra. The characteristic peak at 1720 cm^−1^ corresponds to the stretching vibration of C=O of the ester group. The peak at 1152 cm^−1^ is the stretching vibration peak of C–O–C. The peak at 1454 cm^−1^ is attributed to the vibration of the benzene ring, and the peaks at 757 cm^−1^ and 698 cm^−1^ are assigned to the bending vibration peaks of C-H on the mono-substituted benzene. The multiple peaks at 2800–3000 cm^−1^ are the stretching vibrations of –CH and –CH_2_, whereas a stretching vibration peak of C=C at 1650 cm^−1^ is not observed, indicating a rather high monomer conversion. Moreover, the absorption peak of the epoxy group at around 910 cm^−1^ is not obvious in both cases, which should be attributed to the ring-opening reaction of the epoxy groups and premature crosslinking reaction with the carboxyl groups during the polymerization process [36,37].

### 2.2. Effect of GMA

**Figure 3:**
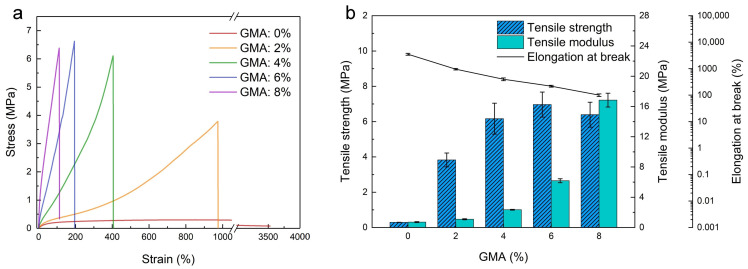
Tensile tests of SA latex films with different GMA dosages. (**a**) Stress–strain curves; (**b**) influences of GMA dosage on the tensile strength, modulus, and elongation at break.

The mechanical properties of the latex films are important since they determine the mechanical performance of EFFTs. Figure 3a depicts the stress–strain curves of the latex films in a uniaxial tensile, showing a brittle fracture except for sample 1 without GMA. Based on Figure 3a, the tensile strength, tensile modulus, and elongation at break are obtained and plotted in Figure 3b. When the GMA dosage increases from 0 to 8%, the tensile strength increases gradually to the highest value at a GMA dosage of 6%, and then levels off, while the tensile modulus rises gradually to 16.13 MPa, and the elongation at break decreases quickly from 3390% to about 100%. This indicates that the formation of a denser crosslinking network structure at high GMA dosages increases the stiffness and brittleness of the films. The highest tensile strength (6.97 MPa) at a GMA dosage of 6% and a decreased tensile strength (6.39 MPa) at a GMA dosage of 8% imply that excessive crosslinking further enhances brittleness and reduces the strength of the film. The main reason for the loss in strength is that the polymer chain segment between the crosslinking points is too short to change the conformation, such that the polymer chain is severely restricted to deform under loading, leading to a premature fracture. As a result, the crosslinking reaction between the epoxy group and the carboxyl group could significantly improve the mechanical robustness and strength of the films. To meet the flexibility and strength requirements of the EFFTs, the suitable GMA dosage should be in the range of 4% to 6%.

**Figure 4:**
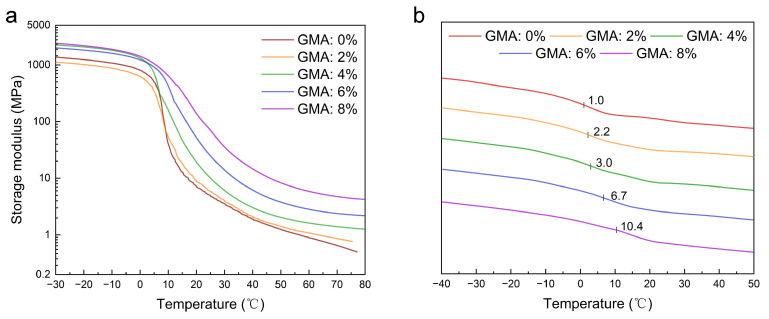
DMA test results and DSC curves of SA latex films. (**a**) DMA test results of SA latex films; (**b**) DSC curves of SA latex films.

The thermomechanical properties of the latex films with different GMA dosages were further determined through DMA to understand the temperature sensibility of the mechanical properties. From the result in Figure 4a, at a low temperature range all of the films are in a glassy state and exhibit a high storage modulus (over 1000 MPa). As the temperature goes up and passes through the glass transition region, the storage modulus decreases sharply to enter the highly elastic state. Thereafter, with an increase in temperature, the storage modulus decreases less and less to reach a higher and higher stable value for films with more and more crosslinking points (from sample 1 to sample 5). This result implies that in the highly elastic state crosslinking can provide films with thermally stable mechanical properties. Moreover, the Tgs of the films can be obtained from the curve point with the highest slope between the glassy state and highly elastic state, which are 7.8 °C, 8.0 °C, 10.0 °C, 14.5 °C, and 18.5 °C for samples 1, 2, 3, 4, and 5, respectively. Because the polymer chain conformation is limited by the crosslinking points, the Tg increases with an increase in the crosslinking density. Moreover, the Tgs of the films were also measured via DSC (see Figure 4b). As the GMA dosage increases from 0 to 8%, the Tg increases gradually from 1.0 °C to 10.4 °C, the same trend to that obtained from DMA curves. In order to endow the EFFT with relatively stable mechanical properties at the service temperature range (10 to 40 °C), the following two aspects should be addressed in designing the molecular structure of the binders: one is that the Tg should be at or near the lower limit service temperature (such as 10 °C), and the other is that densely crosslinked networks should be established among the chains. In this study, the latex films with adequate Tgs and high crosslinking densities were prepared, which should be profitable for forming thermally stable EFFTs.

Water resistance is an important property from a practical viewpoint since high water adsorption will adversely affect the performance of an EFFT. The water absorption of the latex films with different GMA dosages was determined. As shown in Figure S3, the water adsorption first decreases when the GMA dosage increases from 0 to 4%, but it increases at higher GMA dosages (6%, 8%). The lowest water absorption of 7.1% at a GMA dosage of 4% is a rather low value, indicating the good water resistance of the film. In the baking process, latex particles gradually approach each other as the water evaporates, resulting in the deformation and mutual fusion of the particles, followed by entanglement and crosslinking of the molecular chains [38]. As more epoxy groups and carboxyl groups are present in the molecular chains, more crosslinking points could be formed in the baking process, conducive to the low water absorption. However, if there are excessive epoxy groups and carboxyl groups in the molecular chains, highly crosslinked networks would be formed even in the polymerization course (see Figure 2b). As a result, the mutual fusion and post-reaction of the particles would be restricted in the baking process, decreasing the water resistance of the films. The appearances of the absorbent films are also compared in Figure S3; they change from white to slightly blue with an increase in the GMA dosage. Based on the overall film performances, the optimal GMA dosage should be 4% (sample 3).

### 2.3. Effect of AA

It is known that polycarboxylate superplasticizers can be used in building materials because of their high water-reduction capability, which can improve processability and reduce production costs [39,40]. To understand the influence of AA on the properties of the latex films and the processability of the wet mixture formed of latex, mineral powder, and water, another series of SA latices with different AA dosages but without GMA and MAA were prepared (including sample 1 and sample 6 to sample 10; see Table 3) through the emulsion polymerization of AA, BA, and St (the monomer feeding formula is described under Table 3). The viscosity of the latices, the Tgs, and the stress–strain curves of the latex films were determined. From the results in Table 3 and Figure S4, the viscosity of the latices significantly increases as more AA was used in the polymerization process, which is due to the alkali swelling and thickening. The slight increase in Tg, tensile strength, and Young’s modulus with AA dosage should be attributed to the formation of weak hydrogen bonds between the carboxyl groups in the chains. Owing to the linear molecular structure of the polymers, all of the films have a relatively low tensile strength and Young’s modulus, along with high elongation at break (over 1000%; see Figure S4).

The preparation process of an EFFT is outlined in Scheme S1, which typically consists of three steps: (1) mixing mineral powder, SA latex, and deionized water to form a wet mixture (slurry); (2) filling the wet mixture into a mold; and (3) heating the mold with the wet mixture in an oven at a high temperature, followed by demolding.

**Figure 5:**
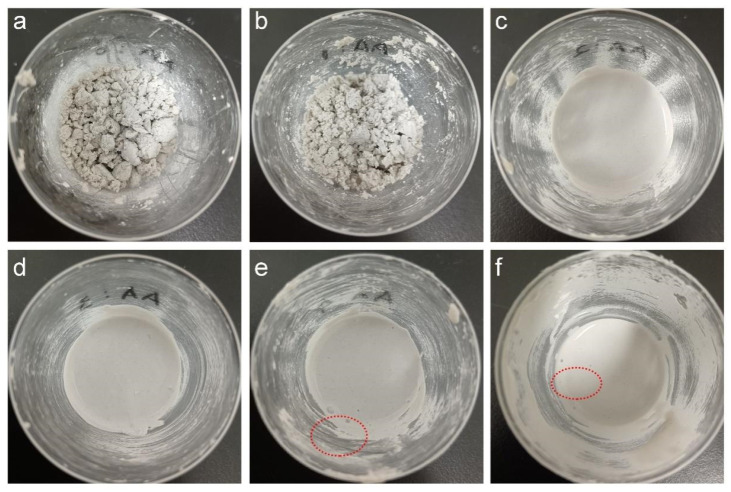
Photographs of the wet mixtures using SA latices with different AA dosages (The red circles indicate the existence of bubbles): (**a**) 0; (**b**) 1%; (**c**) 2%; (**d**) 3%; (**e**) 4%; and (**f**) 6%.

We then investigated the influence of AA dosage on the processability of the wet mixture, which is typically formed from 100 g of mineral powder, 15 g of SA latex, and 20 g of deionized water. As shown in Figure 5, the wet mixtures using different latices present diverse dispersion states, which seriously affect the processability. At AA dosages of 0 and 1%, the mineral powder absorbed the latex and water severely and agglomerated seriously (see Figure 5a,b) to lose mobility. Additionally, 30 g of extra water must be included in the wet mixtures to gain adequate processability for forming an EFFT, while at AA dosages of 2% and higher the mineral powder uniformly dispersed in the wet mixtures with good mobility (see Figure 5c–f). This is because the carboxylic latex particles adsorbed on the surface of the mineral powder provide electrostatic repulsion to drive the mineral powder dispersion and form a hydration layer on the surface of the mineral powder with a lubricating function [41]. This being the case, the carboxyl groups play important roles in the fabrication of an EFFT, including promoting dispersion, reducing water consumption during the mixing process, and shortening the baking time. However, at very high AA dosages (4% and 6%) there are lots of bubbles (indicated by red circles; see Figure 5e,f) in the wet mixtures because of the rather high viscosity of the latices. 

**Figure 6:**
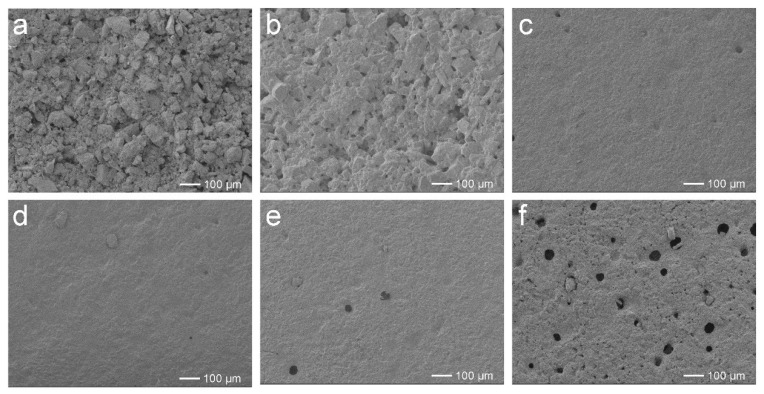
SEM images of EFFTs made by using SA latices with different AA dosages: (**a**) 0; (**b**) 1%; (**c**) 2%; (**d**) 3%; (**e**) 4%; and (**f**) 6%.

The photographs and SEM images of the self-leveling surfaces of EFFTs formed from the wet mixtures are shown in Figure S5 and Figure 6, respectively. At low AA dosages (0 and 1%), 150% more water must be added into the wet mixtures to promote dispersing and processing compared to those of other systems. As a result, the obtained EFFTs had scratches and a large number of small cracks at the surfaces (see Figure S5a,b), which are caused by the volume shrinkage during the baking process. In the corresponding SEM images (see Figure 6a,b) it is observed that the mineral powder particles naturally accumulate together and wide gaps emerge between the particles, suggesting that the obtained EFFTs are quite loose and that the mineral powder particles are easily pulled off from the surface. Increasing AA dosages to 2% and more, the formed EFFTs display quite smooth surfaces (see Figure S5c–f), and the gaps between the powder particles are filled with a polymer binder (see Figure 6c–f) to form a compact microstructure. However, if AA dosages are too high (4% and 6%), air bubbles in the wet mixtures would remain, forming excessive pores in the EFFTs after baking treatment (see Figure 6e,f). Taking into account the processability of the wet mixture and the microstructure of the EFFTs, the optimum dosage of AA should be in the range of 2% to 3%.

**Figure 7:**
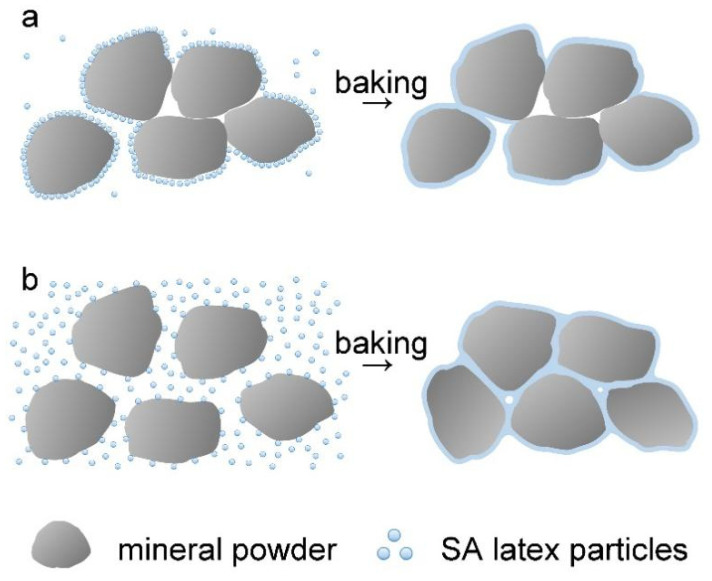
Illustration of the baking process of a wet mixture in the presence of SA latices: (**a**) with low AA dosage; (**b**) with high AA dosage.

To understand the effect of AA on the processability of the wet mixture, the adsorption of latex polymers with AA dosages of 0, 1%, 2%, and 3% on mineral powder was determined by a procedure mentioned in the Materials and Methods section. After centrifugal treatment, the supernatant liquids of the wet mixtures at different amounts of latices were collected and compared, as shown in Figure S6. The supernatant liquids were then dried, and the adsorption of latex polymers on the mineral powder was determined by comparing the weight of latex polymers remaining in the supernatant liquid to that in the original latex. The results shown in Figure S7 indicate that the adsorption percentage of latex polymers on the mineral powder decreases with more AA in polymer chains. On the basis of these results, the effect of AA on the processability of the wet mixture is discussed by using Figure 7. At low dosages (0 and 1%) of AA, the latex particles are adsorbed on the surface of mineral powder particles through the surface active groups (such as hydroxyl groups). The weak repulsion force among different latex particles promotes their accumulation, giving high adsorption of the latex polymer on the mineral powder. After baking treatment, the latex particles on the surface of the mineral powder form a film in situ, resulting in a loose, naturally deposited structure with multiple pores due to the poor mobility of the mineral powders (see Figure 7a). However, at high dosages (2% and more) of AA, the surface of latex particles has more negative charges because of the ionization of the carboxyl groups by ammonia. When the latex is mixed with mineral powder, the latex particles cannot pile up tightly due to the electrostatic repulsion, drastically lowering the adsorption of the latex polymers on the mineral powder particles. In the baking process, as the water evaporates, besides the formation of a film on mineral powder in situ from the absorbed latex polymers, there are also deposition at the surface and cracks of mineral powder from free latex particles in the liquid, conducive to a compact EFFT (see Figure 7b). 

**Figure 8:**
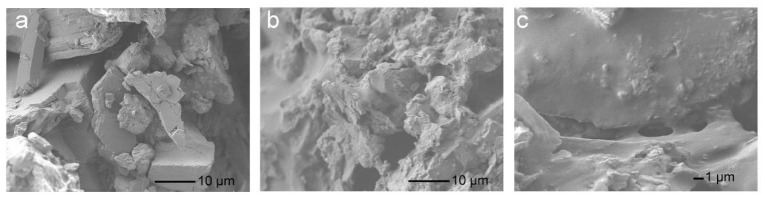
SEM images of the fracture surfaces of EFFTs made by using SA latices with different AA dosages: (**a**) 0 (sample 6); (**b**) 3% (sample 8) at low magnification; and (**c**) 3% (sample 8) at high magnification.

To prove the above suggestion, the mechanical properties of the EFFTs were determined via the three-point bending test, and the fracture surfaces were imaged via SEM (see Figure 8). When sample 6 (without AA) was used as the binder, there were lots of mineral powders unwrapped by the flexible polymer film, along with large gaps between the powder particles in the EFFT sample, as indicated by the SEM image (see Figure 8a). This is because during the preparation of the wet mixture the mineral powders piled up together into large aggregates, with most of the latex polymers at their outer surface; thus, very few latex polymers remained in the internal gaps. When the EFFT is impacted, the lack of effective adhesion between the aggregates would induce the fracture from the weak interface. Because of the obvious cracks, the mechanical properties of the EFFT are too low to be determined via the three-point bending test. In contrast, when using sample 8 (at an AA dosage of 3%) as the binder, most of the powder particles are wrapped by the polymer binder (see the SEM images of Figure 8b,c), indicating that the latex polymer is evenly present at both the surface and the gaps of the mineral powder. The latex polymer inside the gap is beneficial for improving the adhesion between the powders, conducive to the strength of the EFFT. In addition, the mineral powder may form hydrogen bonds and chemical bonds with latex polymers through their active groups, further enhancing the strength [27]. The mechanical property of this EFFT could be successfully evaluated via the three-point bending test, giving the bending strength and bending modulus, which were 0.5 MPa and 20 MPa, respectively.

On the basis of the above results, both a high degree of crosslinking and an adequate content of carboxyl groups are required for the latices to be used as binders of the EFFTs. To meet these requirements, both AA and MAA were polymerized with GMA to prepare the latices (see in Table 1). Because of the different hydrophilic characters, AA and MAA exhibited quite different polymerization behaviors; the more hydrophobic MAA prefers to polymerize inside the particles, whereas the more hydrophilic AA polymerizes in both the aqueous phase and in the particles [42]. The location of the carboxyl groups on the latex particles is also affected by the pH of the reaction medium [42]. In the polymerization course, the pH shifted continuously from about 7.5 to about 4.0 for all samples, further complicating the distribution of the carboxyl groups. Despite this situation, a fraction of MAA would polymerize at the particle surface where AA is preferred to polymerize. Similar to the case of MAA, the hydrophobic GMA is preferred to polymerize inside the latex particles. When a fraction of AA is replaced by MAA, there should be more carboxyl groups inside the latex particles, conducive to the crosslinking reaction between the epoxy group and the carboxyl group. Considering the contribution of MAA, the dosage of AA is settled to 1% in preparing the crosslinking carboxylic SA latices (see Table 1) used as binders for the EFFTs. In such cases, the wet mixtures using these latices (sample 2 to sample 5) could exhibit the desired processability for forming EFFTs with good appearances.

### 2.4. Performances of EFFTs

**Figure 9:**
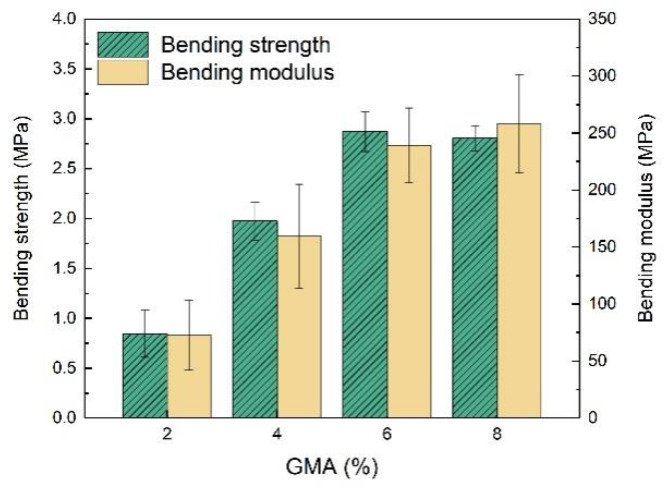
Results of three-point bending tests of EFFTs using latices with different GMA dosages.

The mechanical properties of the resulting EFFTs with different dosages of GMA were determined via the three-point bending test. As shown in Figure 9, both the bending strength and bending modulus of the EFFTs improve as more GMA polymerized into the latices, except for the bending strength at a GMA dosage of 8%, which are basically consistent with those of the latex films (see Figure 3b). Therefore, the mechanical performances of the EFFTs are highly dependent on the polymer binders. If non-crosslinkable latex was used as the binder, the formed EFFT had both a low bending strength and a low bending modulus. For example, the abovementioned EFFT of sample 8 latex had a low bending strength of 0.5 MPa and a low bending modulus of 20 MPa.

**Figure 10:**
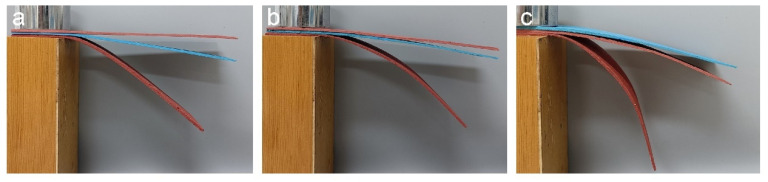
Self-supporting performance of two commercial EFFTs (red) and our EFFT (blue) at different temperatures: (**a**) 0 °C; (**b**) 25 °C; and (**c**) 40 °C.

According to the overall performances of the latex films, the SA latex of sample 3 was selected as the optimal binder to fabricate an EFFT which expressed a series of advantages, including fast curing, a smooth surface, good flexibility, high mechanical strength, and low water absorption (determined to be only 7.4%). By comparing the self-supporting performance at different temperatures, the temperature sensitivity of the EFFT could be understood. Compared with the two commercial EFFTs (red EFFT samples manufactured by Hubei Yaomei Soft Porcelain Co., Ltd., Yichang, China; see Figure 10) made by using ordinary non-crosslinkable SA latices with different hardness values, the EFFT (blue EFFT, see Figure 10) made by using sample 3 latex is more robust and substantially maintains the original shape upon a change in temperature, as it is still flexible at 0 °C and has excellent self-supporting ability even at 40 °C, which should be attributed to the polymer binder. However, both of the two commercial EFFTs (soft and hard) express greater change in shape from 0 °C to 40 °C. This stable self-supporting feature at the whole service temperature range and the very low water absorption suggest that the EFFT made by this novel latex binder should be a promising building decoration material and may have application potential in the construction industry.

## 3. Materials and Methods

### 3.1. Materials

Butyl acrylate (BA), styrene (St), ammonium persulphate (APS), sodium bicarbonate (NaHCO_3_), and ethanol were purchased from Beijing Chemical Works (Beijing, China). Acrylic acid (AA), methacrylic acid (MAA), *N*,*N*-dimethylbenzylamine (BDMA), sodium formaldehydesulfoxylate dihydrate (SFS), and ammonia were obtained from Shanghai Macklin Biochemical Co., Ltd. (Shanghai, China). Glycidyl methacrylate (GMA) was purchased from Aladdin Industrial Co., Ltd. (Shanghai, China). OP-10 was obtained from Jinan Jiehui Chemical Co., Ltd. (Jinan, China). Emulsifier DNS-86 was purchased from Guangzhou Shuangjian Trade Co., Ltd. (Guangzhou, China). Tert-butyl hydroperoxide (TBHPO) and sodium formaldehyde sulfoxylate (SFS) were obtained from Sinopharm Chemical Reagent Co., Ltd. (Shanghai, China). Mineral powder containing mainly calcium powder was obtained from Hubei Yaomei Soft Porcelain Co., Ltd. (Yichang, China). All of the reagents and materials were directly used without further purification.

### 3.2. Preparation of SA Latex

Taking sample 3 as an example, the detailed preparation procedure is described below. Firstly, 0.3 g of DNS-86, 0.3 g of OP-10, and 0.1 g of APS were dissolved in 25 g of deionized water under stirring in a three-necked flask. Under stirring at a rate of 600 rpm, the monomer mixture (31.5 g of BA, 14.8 g of St, 2 g of GMA, 1.2 g of MAA, and 0.5 g of AA) was quickly added dropwise into the water solution. After all of the monomer was added, the mixture was stirred for an additional 30 min. After halting the stirring, a milk-like mixture was obtained, which is defined as the pre-emulsion. Meanwhile, 0.1 g of APS, 0.1 g of NaHCO_3_, and 24 g of deionized water were added into a separate three-necked flask equipped with a mechanical stirrer, a condenser, and a dropping funnel. At a rate of 200 rpm, the mixture was heated to about 60 °C, the 5% pre-emulsion was added to the flask, and then the mixture was further heated to 80 °C for 30 min to prepare the seed emulsion. After this, all of the remaining pre-emulsion was dropped into the mixture via a dropping funnel within 3 h. Subsequently, the polymerization was continued for an additional 1 h. The reaction temperature was then decreased to 60 °C, and a TBHPO solution (0.06 g of TBHPO in 0.5 g of water) as well as an SFS solution (0.04 g of SFS in 0.5 g of water) were dropped into the flask. After reacting at 60 °C for 30 min, the temperature was lowered to 30 °C and suitable amounts of ammonia as well as BDMA (0.5 g of BDMA in 1.5 g of ethanol) were added to obtain the SA latex. 

### 3.3. Preparation of EFFTs

The preparation process of EFFTs is outlined in Scheme S1. A mixture of 100 g of mineral powder, 15 g of SA latex, and 20 g of deionized water was stirred in a 300 mL plastic beaker at a rate of 300 rpm for 5 min to form a wet mixture. After standing for 3 min, 45 g of the wet mixture was filled into a silicone mold (18.5 cm × 6.5 cm × 0.5 cm), and the mold was then slightly vibrated until a self-leveling surface was formed. Next, the mold was placed in an oven preheated at 60 °C for 20 min, and then the temperature was elevated to 100 °C. After being heated for 40 min, an EFFT was prepared after demolding. 

### 3.4. Measurement of the Coagulation Fraction

After polymerization the SA latex was filtered, and the solid residue was collected and dried in an oven at 50 °C for 24 h. The coagulation fraction was calculated through the following equation:

(1) $$\mathrm{coagulation}\;\mathrm{fraction}=\frac{m_{1}}{m_{0}}\;\times \;100\%$$

wherein m_1_ is the weight of the solid residue after drying (g) and m_0_ is the weight of the total monomers fed (g).

### 3.5. Characterization of the SA Latex

After diluting the SA latex to a solid content of about 0.01%, the particle size, polydispersity index (PDI), and Zeta potential of the SA latex particles were determined via a Malvern Mastersizer (3000HS, Malvern, UK). Digital photographs of the appearances of SA latices were taken via mobile phone (Huawei nova 8, Shenzhen, China). The morphology of the SA latex particle was analyzed via scanning electron microscopy (SEM) (JSM7401, Tokyo, Japan) under an acceleration voltage of 3.0 kV. The viscosity of the latex is determined by a viscometer (NDJ-1, Shanghai, China) according to its instruction manual. 

### 3.6. Measurement of the Water Absorption, Swelling Ratio, and Gel Fraction

The initial SA latex film was obtained by drying the wet film (250 μm) in an oven at 100 °C for 45 min. The SA latex film was then removed from the polytetrafluoroethylene panel and soaked in deionized water for 24 h. After this, the SA latex film was taken out and the water on the film surface was wiped using filter paper. The water absorption was calculated based on the following equation:

(2) $$\mathrm{absorption}\;\mathrm{ratio}=\frac{m_{w}{\;-\;m}_{2}}{m_{2}}\;\times \;100\%$$

wherein m_2_ is the weight of the initial latex film (g) and m_w_ is the weight of the latex film after absorbing and wiping (g).

About 0.1 g of the SA latex film was immersed in 100 mL of acetone stirred via magnetic force in a glass cup. After seven days, the swelling film was taken out and the surface solvent was quickly wiped by filter paper and weighted. The swelling film was then dried in an oven at 50 °C for 24 h, and the drying film was weighted. The swelling ratio was calculated via the following equation:

(3) $$\mathrm{swelling}\;\mathrm{ratio}=\frac{m_{s}{\;-\;m}_{3}}{m_{3}}\;\times \;100\%$$

wherein m_s_ is the weight of the swelling film (g) and m_3_ is the weight of the film after drying (g).

The SA latex film with an initial mass of about 0.1 g was wrapped in a filter paper. Another piece of the same filter paper was also wrapped and weighed. The filter paper packages were put in a Soxhlet extractor and extracted with acetone for 12 h. The filter paper packages were taken out and dried in an oven at 50 °C for 6 h. The gel fraction was calculated via the following equation:

(4) $$\mathrm{gel}\;\mathrm{fraction}=\frac{m_{4}{\;-\;(m}_{7}{\;-\;m}_{5}{)\;+\;(m}_{8}{\;-\;m}_{6})}{m_{4}}\;\times \;100\%$$

wherein m_4_ is the initial weight of the SA latex film (g); m_5_ is the initial mass of the latex film and filter paper package (g); m_6_ is the initial mass of the blank filter paper package (g); m_7_ is the mass of the latex film and filter paper package after extracting and drying (g); and m_8_ is the mass of the blank filter paper package after extracting and drying (g).

### 3.7. Characterization of the SA Latex Film

The mechanical properties of the SA latex film were tested under uniaxial tension with a precision universal tester (Shimadzu AGS-X, Kyoto, Japan) using a type 4 dumbbell spline according to GB/T 528-2009 [43], with a tensile speed of 50 mm/min. A dynamic mechanical analysis (DMA) (Q800, New Castle, DE, USA) test was developed at a frequency of 1 Hz, a strain of 1%, a temperature range of −80 to 80 °C, and a heating rate of 5.0 °C/min. The glass transition temperature (Tg) was measured via a differential scanning calorimeter (DSC) (DSC 214 Polyma, Selb, Germany) at a heating rate of 20 °C/min and a temperature range of −80 °C to 60 °C.

### 3.8. Adsorption Characterization of Polymer on the Mineral Powder

The initial adsorption isotherms of the latices with different dosages of AA on the mineral powder were measured with increasing amounts of 5%, 10%, 15%, 20%, and 25%. Firstly, 1 g of the mineral powder, 3 g of deionized water, and the SA latex were mixed and stirred at a rate of 300 rpm for 5 min. Then, 2 g of the wet mixture was taken out and centrifuged for 5 min at 3000 rpm. Then, 1.5 g of the supernatant liquid was collected and dried. The adsorption percentage of the latex particles on the mineral powder could be determined by comparing the weight of polymers in the supernatant liquid and that of the original latex.

### 3.9. Characterization of EFFTs

The morphology and microstructure of EFFTs were analyzed via scanning electron microscopy (SEM) (JSM7401, Tokyo, Japan) under an acceleration voltage of 3.0 kV. The bending mechanical properties of EFFTs were measured via a universal tester (UTM-1432, Chengde, China), with a bending rate of 10.0 mm/min and a sample span of 80.0 mm. 

## 4. Conclusions

This study investigated the preparation of crosslinking carboxylic SA latices and their application as binders to form thermally stable EFFTs. The main conclusions drawn are as follows: Crosslinking carboxylic SA latices with high stability and uniform particles were successfully prepared via semicontinuous seed emulsion polymerization of GMA, MAA, AA, BA, and St. Latex films with a high degree of crosslinking could be formed based on the reaction between the epoxy group and the carboxyl group catalyzed by BDMA at 100 °C. At a GMA dosage range of 4% to 6%, the latex films exhibited both high water resistance and excellent mechanical properties. DMA analysis indicated that at the highly elastic state the crosslinking film had more stable mechanical properties compared to the non-crosslinkable film as the temperature changed, which was desired for forming thermally stable EFFTs. The influences of AA dosage on the processability of the wet mixture and the microstructure of EFFTs were further explored and discussed. The results indicated that the optimum dosage of AA should be in the range of 2% to 3%, in which the wet mixtures exhibited adequate processability for forming EFFTs with compact microstructures and high adhesion. Owing to the excellent properties of the latex film, the obtained EFFT exhibited a series of improved properties, including fast curing, smooth surface, good flexibility, high mechanical properties, and low water absorption. In particular, the self-supporting ability over a wide temperature range implied that this EFFT could be applied in diverse climate conditions, showing the application potential in the decoration and construction industries. Further study of the long-term durability of the EFFT using the present SA latex is advised to propose suitable application aspects of such materials.

## Figures and Tables

**Table 1:** Monomer feeding formula of the SA latices.

Sample	GMA (%)	AA (%)	MAA (%)	Epoxy Group/Carboxyl Group ^1^	BA (%)	St (%)
1	0	1	0	---	65	34.0
2	2	1	0.2	1:1.14	65	31.8
3	4	1	1.4	1:1.08	65	28.6
4	6	1	2.6	1:1.04	65	25.4
5	8	1	3.8	1:1.03	65	22.2

^1^ The stoichiometric ratio of the epoxy group and the carboxyl group.

**Table 2:** Influences of GMA dosage on coagulation fraction, particle size, and Zeta potential of the latices.

Sample	GMA (%)	Coagulation Fraction (%)	Particle Size (nm)	PDI	Zeta Potential (mV)
1	0	0.213	369	0.111	−50.2
2	2	0.104	342	0.100	−46.7
3	4	0.069	309	0.172	−45.8
4	6	0.108	299	0.156	−48.0
5	8	0.137	334	0.161	−47.6

**Table 3:** Influences of AA dosage on the properties of SA latices and SA latex films.

Sample	AA ^1^ (%)	Viscosity (CPS)	Tg (°C)	Tensile Strength (KPa)	Tensile Modulus (KPa)
6	0	23.5	−8.3	320	419
1	1	31.2	−7.8	381	403
7	2	73.7	−7.4	492	449
8	3	1.90 × 10^3^	−7.3	564	467
9	4	1.71 × 10^4^	−7.2	678	478
10 ^2^	6	Very high	-	-	-

^1^ Besides the dosage of AA, the dosage of BA is settled to 65%, and the dosage of St is calculated by subtracting the dosages of AA and BA from 100%. ^2^ The viscosity of the latex of sample 10 is too high to form a film.

## Data Availability

Not applicable.

## Supplementary Materials

The following supporting information can be downloaded at: https://www.mdpi.com/article/10.3390/molecules28176249/s1, Figure S1: SEM image of latex particles of sample 3 and particle size measurement using Nano Measure 1.2.0 software; Figure S2: FT-IR spectra of sample 5 before and after baking; Figure S3: Influence of GMA dosage on the water absorption of the latex films; Figure S4: Influence of AA dosage on the mechanical properties of the latex films; Scheme S1: The process of preparing the EFFT; Figure S5: Photographs of the EFFT using SA latices with different AA dosages: (a) 0; (b) 1%; (c) 2%; (d) 3%; (e) 4%; and (f) 6%; Figure S6: Supernatant liquid of the wet mixture using different latices as the binders; and Figure S7: Adsorption percentage of SA latex particles with different AA dosages on the mineral powder.
