# Supplementary material for: Fabrication of Novel Crosslinking Carboxylic Styrene-Acrylate Latices as Binders for Exterior Flexible Facing Tiles
Source: Molecules. 2023 Aug 25;28(17):6249. doi: 10.3390/molecules28176249 (PMC10488960; doi:10.3390/molecules28176249)
Supplement: Supplementary file 1 [file molecules-28-06249-s001.zip › molecules-2522927-supplementary.pdf]

Supporting information

Fabrication of novel crosslinking carboxylic styrene-acrylate latices as binders for exterior flexible facing tiles

Yue Lu, Jing-Ke Wei, Hao- Jie Jin, Li-Ming Tang*

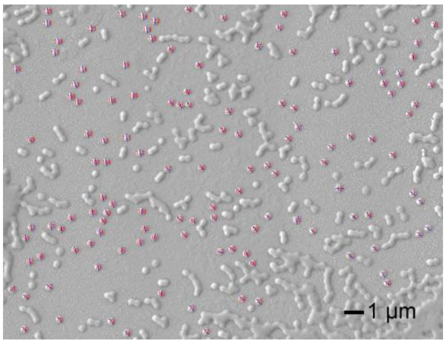

Figure S1. SEM image of latex particles of sample 3 and the particle size measurement using Nano Measure 1.2.0 software.

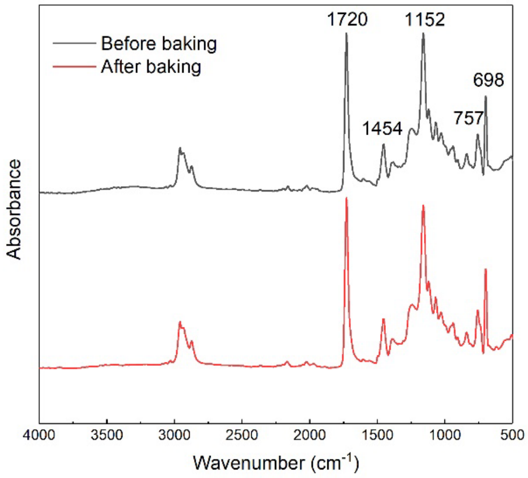

Figure S1. FT-IR spectra of sample 4 before and after baking.

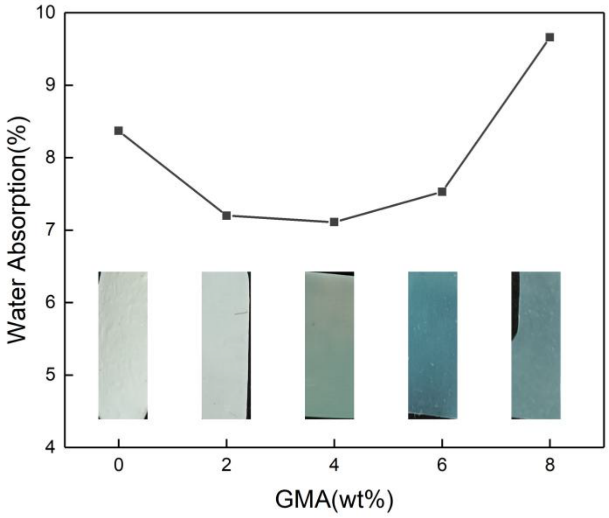

Figure S3. Influence of GMA dosage on water absorption of the latex films.

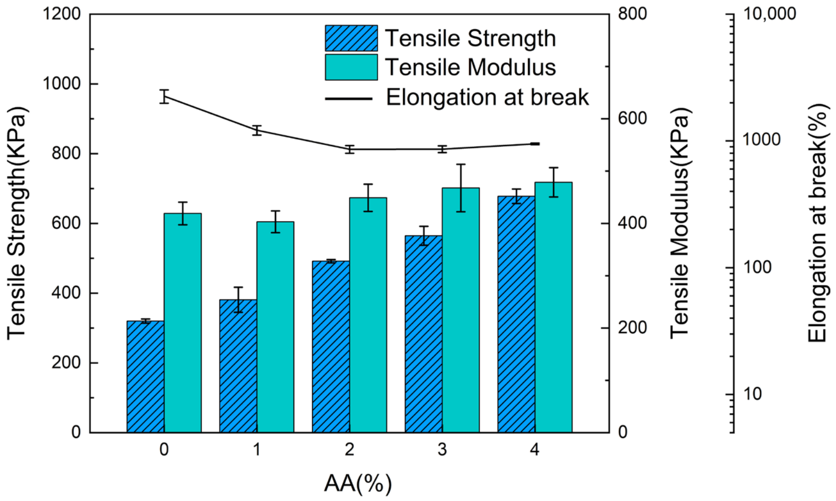

Figure S4. Influence of AA dosage on the mechanical properties of the latex films.

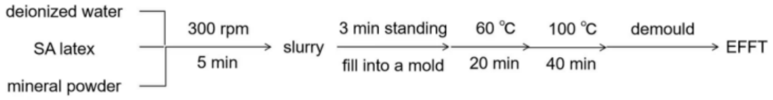

Scheme S1. The process of preparing EFFT.

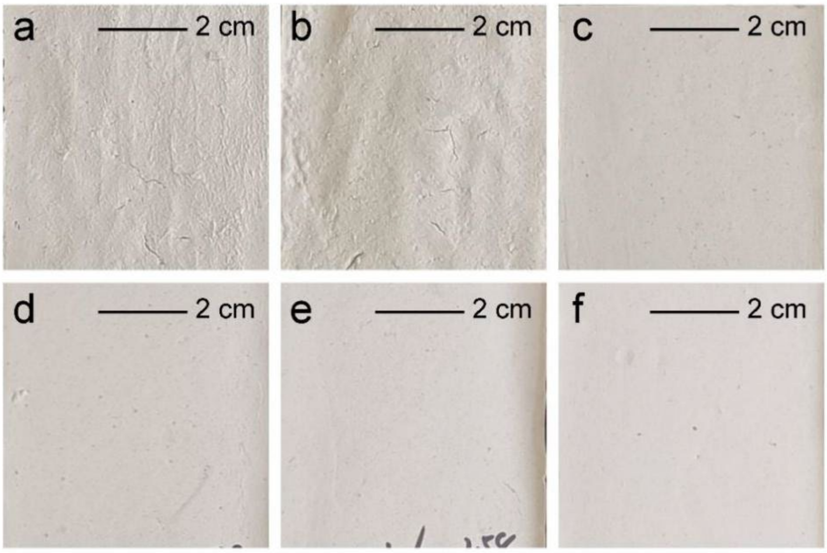

Figure S5. Photographs of EFFT using SA latices with different AA dosages:

(a) 0; (b) 1%; (c) 2%; (d) 3%; (e) 4%; (f) 6%.

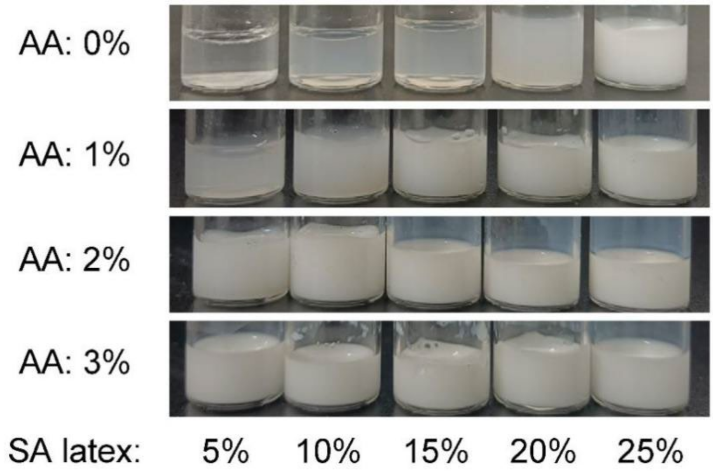

Figure S6. Supernatant liquid of wet mixture using latices with different AA dosages.

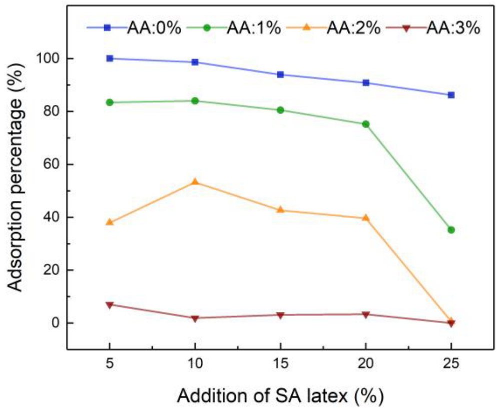

Figure S7. Adsorption percentage of SA latex particles with different AA dosages on the mineral powder.
